# The impact of farm practices and wild carriers on white spot disease in marine shrimp in Rayong Province, Thailand

**DOI:** 10.14202/vetworld.2023.111-117

**Published:** 2023-01-16

**Authors:** Sompit Yaemkasem, Jiraporn Promchairat, Pinchakorn Srithongkhum, Napawan Paungsroy, Chaithep Poolkhet

**Affiliations:** 1 Rayong Coastal Aquaculture Research and Development Center, Rayong, 21000, Thailand; 2 Petchabun Inland Aquaculture Research and Development Center, Petchabun, 67000, Thailand; 3 Department of Veterinary Public Health, Faculty of Veterinary Medicine, Kasetsart University, Kamphaeng Saen Campus, Nakhon Pathom, 73140, Thailand

**Keywords:** disease carrier, farm practices, water management, white spot disease

## Abstract

**Background and Aim:**

White spot disease (WSD) is a highly lethal and contagious viral disease in marine shrimp caused by the white spot syndrome virus (WSSV). White spot disease impacts the worldwide crustacean aquaculture sector, including Thailand. This study aimed to investigate the effect of farm management practices and wild carriers on WSD occurrence in grow-out marine shrimp farms in Rayong Province, Thailand.

**Materials and Methods:**

A longitudinal study was conducted using a structured questionnaire from June 2018 to June 2020. A total of 186 questionnaires for 186 ponds were collected from 15 shrimp farms. Univariate and multivariable analyses using generalized estimating equations were used to determine the risk factors associated with WSD. In addition, possible carrier samples (wild shrimp and wild crabs) were collected inside and outside farms to test for the presence of WSSV.

**Results:**

Direct discharge of treated wastewater into farm ponds was statistically significant in the final model (p < 0.01), with an odd ratio (OR) factor of 0.097 (95% confidence interval [CI] of OR = 0.007–0.242). Pooled sampling for WSSV in wild shrimp and crabs showed that 48 out of 936 (5.13%) samples tested positive for WSD using nested polymerase chain reaction. The samples from banana shrimp, jinga shrimp, banded snapping shrimp, dwarf prawn, whiteleg shrimp, green tidal crabs, and mangrove crabs tested positive.

**Conclusion:**

Based on the findings of this study, we infer that the environment plays an important role in the spread of this disease. The results of this study will provide insights into the effective planning of disease control.

## Introduction

White spot disease (WSD) is a highly lethal and contagious viral disease in marine shrimp that is caused by the white spot syndrome virus (WSSV). This double-stranded DNA virus is assigned to the genus *Whispovirus*, which belongs to the family *Nimaviridae* [1]. White spot syndrome virus is an extremely virulent pathogen in cultured shrimp that causes 100% mortality within a few days of an outbreak in normal culture conditions [2]. White spot syndrome virus can infect a wide range of aquatic crustaceans, including marine shrimp and crabs [3]. White spot disease impacts the worldwide crustacean aquaculture sector [4, 5]. The previous study has reported an estimated loss of approximately 1 billion USD and a 15% reduction in global shrimp production due to WSD [6].

Many risk factors for WSD and its outbreaks have been identified. For example, Corsin *et al*. [7] reported an association between farm management practices and outbreaks of WSD in ponds located close to the sea. The use of water sources shared with other farms [8] and wastewater from processing plants [9] are considered potential risk factors for WSD. However, filtering the water before filling up culture ponds will prevent the entry of species that could be disease carriers [10]. Researchers have reported that in Thailand, sourcing water from communal canals, culturing shrimp year-round, a single owner operating more than one farm, the presence of WSD in previous crops, and the use of seawater were associated with WSD [11, 12]. Studies in Rayong Province, Thailand, between October 2015 and September 2018 reported significant disease clustering in ponds near the sea [13]. In addition to these risk factors, possible changes in the spatial effects and temporal distributions in each area of the farming system also require more studies to plan disease management. Although wild animals are known carriers of WSD, there are no epidemiological reports of their association with WSD in ponding sites in Thailand. An experimental study has shown that three species of crab (*Sesarma* spp., *Scylla serrata*, and *Uca pugilator*) act as carriers of infection [14]. The injection of WSSV into three crustaceans (the sand crab *Portunus pelagicus*, the mud crab *S. serrata*, and the krill *Acetes* spp.) led to all the krill dying in 3 days, while sand and mud crabs showed 100% and 20% mortality in 8 and 9 days, respectively [14, 15]. Soowannayan and Phanthura [16] reported the transmission of the virus to penaeid shrimp by WSSV-infected red claw crayfish (*Cherax quadricarinatus*). Thus, finding infected vectors in shrimp farms is an important step in planning effective disease control.

Rayong Province is in the eastern part of Thailand and is connected to the Gulf of Thailand. This province is an important intensive shrimp farming area in Thailand that is also an endemic area of WSD. This study aimed to discover the most effective way to control the disease, this study investigated the effects of farm management practices and wild carriers on WSD occurrence in grow-out marine shrimp farms in Rayong Province, Thailand, to improve farm biosecurity and control of WSD.

## Materials and Methods

### Ethical approval and Informed consent

The study was approved by the Department of Fisheries committee for ethical considerations in animal usage (Approval no. U1-05341-2559 l5).

### Study period and location

A longitudinal study was conducted using a structured questionnaire from June 2018 to June 2020 involving 15 volunteer marine shrimp farms in Rayong Province. Observations were recorded based on the owners’ convenience at the farms in Muang District (n = 3) and Klaeng District (n = 12) without interfering with their work.

### Study framework, location, WSD status, and ethical statement

In this study, the unit of interest was the pond at each farm. In this regard, each farm was studied for one to seven sequential pondings that might have overlapped with the previous or subsequent cultivations. Before ponding, all batches of post-larvae (PL) were prechecked for WSD, acute hepatopancreatic necrosis disease, and *Enterocytozoon hepatopenaei* by the local laboratory of the DOF, Thailand, and tested negative before release.

Most shrimp farms in Rayong Province are located near mangrove forests. Farms near the Gulf of Thailand usually receive water from the sea or canal directly, and it is, therefore, likely that wild crabs, wild shrimp, or birds appear in water or farm areas. However, farmers usually take the necessary biosecurity measures to prevent disease transmission from wild animals to cultivated shrimp, such as protective fences, pond linings, bird protection, and water filtration systems.

**Figure-1:**
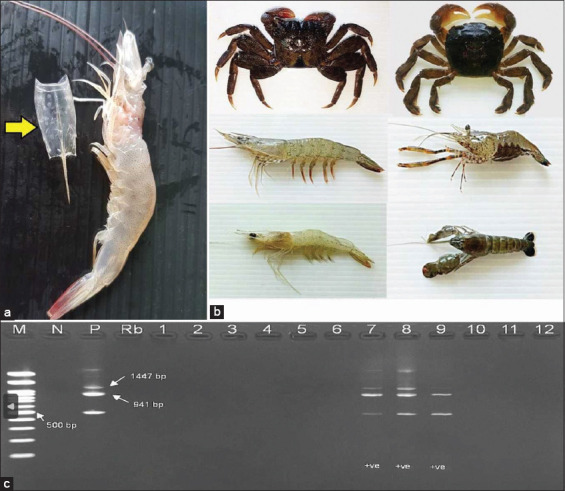
Infected whiteleg shrimp (a) and some infected carriers (b) of white spot disease (c) confirmation of white spot disease virus using gel electrophoresis of 1,447 and 941 bp nested polymerase chain reaction amplicons. Positive samples are shown in columns 7–9.

The DOF protocol was used to confirm WSD status during ponding. Observed symptoms included moribund shrimp or living shrimp with white spots on the exoskeleton (Figure-1) and a reddish body discoloration, the presence of floating shrimp around the edges of the pond surfaces, a decrease in food consumption, and a surge in the mortality rate. Shrimp suspected to have WSD are first tested by farmers using WSSV strip test kits (EnBiotech Laboratories; sensitivity = 34.7%, specificity = 100%). However, for all suspected WSD ponds, the farmer must inform the local DOF and bring at least 15 suspected shrimps to the local DOF laboratory. A nested polymerase chain reaction (PCR) is used to confirm the disease. In the case of the absence of suspected WSD shrimp before harvest, 60 normal shrimps collected at harvest were checked for WSD using nested PCR [17, 18].

### Data collection and questionnaire

Using a questionnaire, data on the activities of 15 target shrimp farms were collected on the day of the visit. The questionnaire was designed based on previous reports [11, 12, 17] and the authors’ own field experiences.

The questions focused on farm management, water management, and carrier control, including the main risk factors of WSD. The questionnaire contained both open and closed questions, and face-to-face interviews were also conducted with the respondents. The questionnaire was verified by aquaculture and epidemiology experts and initially tested on 20 farmers to determine whether any corrections were required. The corrected questionnaire was then discussed with local fishery officers, who collected the data. In this study, all respondents were farm owners or managers.

During the production cycle, depending on the question, farmers were interviewed twice on stocking and harvesting days during cropping. To prevent recollection bias, questions addressing the source of the PL, pond preparation, water preparation, biosecurity, and the appearance of wild carriers were asked on stocking day. On harvesting day, all remaining questions were asked, such as those concerning the occurrence of wild carriers during cultivation, water management, feed management, human control, farm practices, farm environment, and disease status. Farmers were also asked about any use of other techniques or activities that might have caused a WSD outbreak — for example, any biosecurity measures they forgot to follow.

### Carriers

Possible carrier samples (wild shrimps and wild crabs) were collected inside and outside the farms using the convenience sampling method. In each sampling, five carriers from each species were pooled as one sample. In this study, the sites of carrier collection were divided into two categories: (1) Inside the farms and surrounding areas, and (2) outside the farms. Inside the farms and the surrounding areas, possible carrier samples were collected at night in reservoirs, inlet canals, drainage ponds, and communal canals (15 stations × 3 seasons × 60 carriers = 2,700/5 = 540 pooled samples). The specimens sampled were banana shrimp (*Fenneropenaeus merguiensis*, n = 21), jinga shrimp (*Metapenaeus affinis*, n = 16), banded snapping shrimp (*Alpheus euphrosyne*, n = 2), dwarf prawn (*Macrobrachium equidens*, n = 53), whiteleg shrimp (*Litopenaeus vannamei*, n = 12), green tidal crabs (*Varuna yui*, n = 208), and mangrove crabs (*Episesarma* spp., n = 228). Outside the farms, possible carriers were collected from water sources along the coastline of Rayong Province (11 stations × 3 seasons × 60 carriers = 1980/5 = 396 pooled samples). The samples consisted of banana shrimp (n = 9), Jinga shrimp (n = 8), dwarf prawn (n = 25), whiteleg shrimp (n = 1), green tidal crabs (n = 187), and mangrove crabs (n = 166). In total, 936 pooled samples were sent to a DOF laboratory for WSD detection using nested PCR.

### Laboratory testing

All shrimp samples and carriers were tested using nested PCR [17−20]. The primer sets 146F1 (5´ ACTACTAACTTCAG CCTATCTAG 3´) /146R1 (5´TAATGCGGGTGTAAT GTTCTTAC GA 3´) and 146F1/146R1 plus 146F2 (5 ´GTAACTGCCCCTTCCATCTCCA 3´)/146R2 (5´ TACGGC AGCTGCTGCACCTTGT 3´), respectively, were used for the first and nested steps. The PCR mixture for both reactions consisted of a DNA-free water 2.20 μL/sample, a 2× PCR master mix 5 μL/sample (Sigma), a 10 μM W146F1 primer 0.2 μL/sample, a 10 μM W146R1 primer 0.2 μL/sample, a 5 μM EF1aS-F 0.2 μL/sample, and a 5 μM EF1aS-R 0.2 μL/sample. The cycling conditions for the first step assays were 94°C for 3 min 1 cycle, 94°C for 30 s, 60°C for 30 s 15 cycles, 72°C for 30 s, and a final extension step at 72°C for 3 min 1 cycle. In the first step reaction, 10 μL was used as a template for the nested PCR. The cycling conditions for nested assays were 94°C for 3 min 1 cycle, 94°C for 30 s, 60°C for 30 s 30 cycles, 72°C for 30 s, and a final extension step at 72°C for 3 min 1 cycle. The products were visualized in 1.5% agarose gel electrophoresis containing 2 μL SERVA DNA gel stain. The expected amplicon sizes were 1447 and 941 bp, respectively, for the first and nested step reactions (Figure-1). In addition, the epidemiological sensitivity and specificity of this nested PCR were 97.3% and 100%, respectively [15].

### Statistical analysis

Univariate and multivariable analyses using generalized estimating equations (GEEs) were used to determine the risk factors associated with WSD using the R package [21] ”geepack” [22, 23]. The quasi-information criterion (QIC) function was used for model selection [24]. In addition, ArcGIS release 10.8.2 (ESRI, Redlands, California, USA) was used for mapping.

## Results

### General information

A total of 186 questionnaires for 186 ponds were collected from 15 shrimp farms. Most farms (98.39%) raised a monoculture of whiteleg shrimp (183/186), and only 1.61% (3/186) of ponds raised a monoculture of black-tiger shrimp (*Penaeus monodon*). Most farms were under cultivation with small pondings. It was found that 10 farms (66.67%) contained two to five active ponds. The remaining four farms (26.67%) contained 6–10 active ponds, and one farm (6.67%) contained more than 10 active ponds. The PL was from Trad, Chachoengsao, Chonburi, and Rayong. During the study, the median price for PL was 0.185 baht (minimum–maximum: 0.08–0.50) per larvae. Farmers began raising the shrimp at PL 12 and median stage PL 13.5 (minimum–maximum: 12–36). For farm biosecurity, 4/15 (26.67%) farms used a fence, 10/15 (67%) used a bird net, 6/15 (40%) used a crab fence, 14/15 (93.33%) used a water filter, and 13/15 (86.67%) used a water disinfectant. Only one (6.67%) farm used all the control measures — that is, disinfecting vehicle tires, using bird nets and crab fences, and disinfecting people before they entered the farms. The managers of 6/15 farms (60%) were responsible for multiple farms. Only one farm (6.67%) lacked a reservoir for water stocking, water filtration, and wastewater management.

### Univariate and multivariable analysis

For univariate analysis, three variables were statistically associated with WSD occurrence (p < 0.05; Table-1): The lack of a reservoir pond, addition of water into ponds during the stocking period, and direct discharge of wastewater after proper treatment. On further analysis of the three variables using multivariable GEE, one variable was found to be statistically significant in the final model (p < 0.01). Directly discharging wastewater after proper treatment (Table-2) yielded an odd ratio (OR) of 0.097 (95% confidence interval [CI] of OR = 0.007–0.242). In contrast, farms directly discharging wastewater into natural resources without proper treatment were at a 10.309 (1/0.097) times greater risk of WSD occurrence than those that discharged water after proper treatment. In addition, the QIC value of the final model was 201.12 using exchangeable covariance, and the final model was also calculated using independent covariance (QIC = 207.24). We found that the QIC for the exchangeable term was lower than that of the independent term. This indicates that the final model registered a lower correlation structure value.

**Table-1:** Univariate analysis of risk factors for white spot disease in marine shrimp farms in Rayong, Thailand.

Factors	Number of infected ponds (%)	Number of non-infected ponds (%)	p-value
Lack of a reservoir pond (reference = no)	44 (23.65)	123 (66.13)	0.01
Have a reservoir	3 (1.61)	16 (8.60)	
Addition of water during stocking period (reference = yes)	29 (15.59)	113 (60.75)	0.01
None	18 (9.67)	26 (13.98)	
Direct discharge of wastewater with Proper treatment (reference = no)	19 (10.21)	16 (8.60)	< 0.01
Yes	28 (15.05)	123 (66.13)	

**Table-2:** Final model of risk factors for white spot disease in marine shrimp farms in Rayong, Thailand. The correlation structure is exchangeable.

Factors	Estimate	SE	OR	95% CI	Wald	p-value
Intercept (exchangeable term)	0.648	0.667	-	-	0.94	0.33
Direct discharge of wastewater after Proper treatment (reference = no)	-	-			-	-
Yes	−2.336	0.563	0.097	0.007–0.242	17.24	< 0.01

SE=Standard error, OR=Odds ratio, CI=Confidence interval, Quasi-information criterion (QIC) of exchangeable term=201.12, QIC of independence term=207.24

### Detection of WSSV in carriers

**Figure-2:**
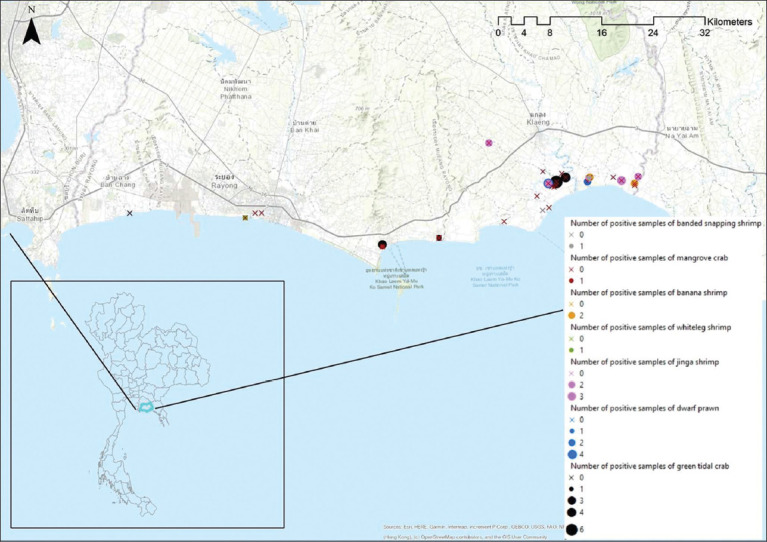
Map of Thailand and Rayong Province showing sampling sites and positive sampling sites associated with the white spot syndrome virus in wild carriers [Source: Map generated by ArcGIS 10.8.2 software].

As per nested PCR, 48 of the 936 (5.13%) pooled samples tested positive for WSD. Inside the farms and surrounding areas, it was found that 26 of the 540 (4.81%) samples had positive WSD results, including banana shrimp (2/21; 9.52%), jinga shrimp (13/16; 81.25%), banded snapping shrimp (1/2; 50%), dwarf prawn (7/53; 13.21%), whiteleg shrimp (1/12; 8.33%), and green tidal crabs (2/208; 0.96%), but no mangrove crabs (0/228; 0%). Outside the farms, 22 of the 396 (5.56%) samples were positive for WSD, including banana shrimp (2/9; 22.22%), jinga shrimp (2/8; 25%), dwarf prawn (4/25; 16%), whiteleg shrimp (0/1; 0%), green tidal crabs (13/187; 6.95%), and a mangrove crab (1/166; 0.60%). The appearances of some infected carriers in this study are shown in Figure-1. In addition, the locations of the infected carriers are shown in Figure-2.

## Discussion

In this study, the final GEE model showed that the direct discharge of wastewater after proper treatment was the only factor associated with WSD occurrence on shrimp farms. Possible carriers for WSSV were detected both inside farms and the surrounding areas and outside farms. Using nested PCR, it was found that 5.13% of wild shrimp and crabs tested positive for WSSV. The percentages of positive WSSV samples from inside farms and the surrounding areas and outside farms were 4.81 and 5.56, respectively.

The study also revealed that the direct discharge of wastewater after proper treatment protected against WSD. However, the release of wastewater without prior treatment into natural resources by several farmers resulted in the possible spread of WSSV and subsequent circulation in the farming area. This calls for the relevant authorities to educate farmers about exercising caution on this issue. The previous study [25] has also shown that the virus is readily transmitted from diseased to healthy susceptible shrimp through contaminated water. Using sodium hypochlorite, which releases free chlorine in the water (> 100 ppm; 10 min) [1], and retaining this water within the farm can also reduce the spread of the disease. In addition, a quick response aids in the prevention of disease spread to other ponds in the same farm, other farms in the region, or the natural environment. Furthermore, the sharing of water sources between farms poses a risk and is strongly correlated with the presence of WSD in adjacent farms [26]. Discharged water can lead to contamination, both on the farm where the water is discharged and on other farms [8]. Drawing water from communal canals, culturing shrimp year-round, and a single owner operating more than one farm are risk factors for WSD [11]. Contamination can also come from sludge removed from the bottoms of ponds [27]. These facts indicate that the introduction or release of water from a farm is critical in preventing diseases, and that farmers must keep a strict vigil at all times. However, it was found in this study that factors related to the introduction of water for ponding were not statistically significant. Hence, it is concluded that farmers during the study period in this area might have neglected other issues related to the discharge of wastewater from their farms.

Based on our field observations, winds and storms can transport water contaminated with WSSV from the sea to nearby shrimp farms, increasing the risk of WSD occurrence. In this study, it was also found that many farmers did not follow protective measures, such as building fences to protect against possible infected carriers from outside the farms during ponding, making the farms more vulnerable to WSD infection. In this regard, relevant agencies must instruct farmers to find ways to mitigate the risk. In addition, we found that the percentage of positive WSSV samples from inside the farms and surrounding areas and outside the farms were similar. This phenomenon demonstrates that biosecurity measures to control or prevent the appearance of carriers within a farm are one of the key measures for reducing the chances of disease transmission from exposure to infected carriers.

Our study showed that positive wild shrimp samples were more common than positive crab samples, which contrasts with a prior observation that WSSV infections were more common in wild crabs than in wild shrimp [28]. This may be due to spatial heterogeneity. However, positive results for WSSV in wild shrimp and crabs indicated that WSSV circulates in the environment, both inside and outside farms. Therefore, environmental protection measures to prevent the spread of disease in farms are important. In addition, the reduction of pathogens in natural resources is the responsibility of the relevant authorities and farmers and is consistent with the risk factors reported in this study. However, due to budget limitations, the authors were unable to determine the DNA-level correlation between WSSV-positive samples in cultured shrimp and carriers. This aspect will be investigated in future studies.

A few reports from Thailand Provide evidence of WSSV infection from wild carrier samples collected from natural resources. Hamano *et al*. [29] detected WSSV in wild *P. monodon* in another province of Thailand. This may indicate that wild WSSV-infected shrimp commonly appear in Thailand’s environment. In terms of temporal variation, the relevant authorities should set up a surveillance system to study and understand the prevalence of WSD in wild carriers and the genetic variations of WSSV. This will provide important information on disease transmission, which can help us implement more effective disease control. Therefore, this study emphasizes the importance of reporting the major risk factors and the presence of WSSV in natural carriers.

## Conclusion

The direct discharge of wastewater after proper treatment was the only statistically significant factor associated with WSD occurrence. Among wild carriers, those that tested positive for WSSV were banana shrimp, jinga shrimp, banded snapping shrimp, dwarf prawn, whiteleg shrimp, green tidal crabs, and mangrove crabs. The results of this study will be useful in planning disease control.

## Authors’ Contributions

SY and CP: Study design, data analysis, and drafted the manuscript. SY, JP, PS, and NP: Data collection and laboratory work. All authors have read and approved the final manuscript.
